# Ammonia‐oxidizing archaea release a suite of organic compounds potentially fueling prokaryotic heterotrophy in the ocean

**DOI:** 10.1111/1462-2920.14755

**Published:** 2019-08-06

**Authors:** Barbara Bayer, Roberta L. Hansman, Meriel J. Bittner, Beatriz E. Noriega‐Ortega, Jutta Niggemann, Thorsten Dittmar, Gerhard J. Herndl

**Affiliations:** ^1^ Division of Bio‐Oceanography, Department of Limnology and Bio‐Oceanography, Centre of Functional Ecology University of Vienna Vienna 1090 Austria; ^2^ International Atomic Energy Agency – Environment Laboratories Radioecology Laboratory, 98000 Monaco Monaco; ^3^ ICBM‐MPI Bridging Group for Marine Geochemistry University of Oldenburg, 26129 Oldenburg Germany; ^4^ Department of Marine Microbiology and Biogeochemistry NIOZ Royal Netherlands Institute for Sea Research, and Utrecht University, 1790 AB Den Burg, Texel The Netherlands

## Abstract

Ammonia‐oxidizing archaea (AOA) constitute a considerable fraction of microbial biomass in the global ocean, comprising 20%–40% of the ocean's prokaryotic plankton. However, it remains enigmatic to what extent these chemolithoautotrophic archaea release dissolved organic carbon (DOC). A combination of targeted and untargeted metabolomics was used to characterize the exometabolomes of three model AOA strains of the *Nitrosopumilus* genus. Our results indicate that marine AOA exude a suite of organic compounds with potentially varying reactivities, dominated by nitrogen‐containing compounds. A significant fraction of the released dissolved organic matter (DOM) consists of labile compounds, which typically limit prokaryotic heterotrophic activity in open ocean waters, including amino acids, thymidine and B vitamins. Amino acid release rates corresponded with ammonia oxidation activity and the three *Nitrosopumilus* strains predominantly released hydrophobic amino acids, potentially as a result of passive diffusion. Despite the low contribution of DOC released by AOA (~0.08%–1.05%) to the heterotrophic prokaryotic carbon demand, the release of physiologically relevant metabolites could be crucial for microbes that are auxotrophic for some of these compounds, including members of the globally abundant and ubiquitous SAR11 clade.

## Introduction

Dissolved organic matter (DOM) is one of the Earth's largest reactive carbon pools, similar in magnitude to atmospheric CO_2_, and of major significance for the global carbon cycle and climate (Hedges, 1992; Hansell *et al*., 2009). DOM is highly complex, consisting of thousands of different organic molecules (Kim *et al*., 2003; Hansman *et al*., 2015), and comprising the most heterogeneous and dynamic pool of carbon in the oceans (Kujawinski *et al*., 2009). Its concentration and composition are affected by biotic processes such as photosynthesis and heterotrophic metabolism (Azam *et al*., 1983; Carlson, 2002), as well as by photochemical processes (Mopper *et al*., 1991). However, while the flux of carbon through this pool of molecules is mediated largely by microbial activity, the relationships between marine microbes and the molecules making up the DOM pool remain poorly characterized (Moran *et al*., 2016).

DOM is released as a by‐product of metabolically active microbes, but may also be released for nutrient acquisition and communication (in the forms of metal‐binding ligands and quorum sensing chemicals, respectively) (Carlson, 2002; Gram *et al*., 2002; Vraspir and Butler, 2009), as well as upon predation or viral lysis (Nagata, 2000). The fraction of DOC exuded by phytoplankton is highly variable, and accounts on average for ∼5% to 50% of photosynthetically fixed carbon, whereas heterotrophic bacteria have been shown to release ∼1%–9% of their produced DOC (Carlson, 2002). There are major gaps, however, in our understanding of the biogeochemical significance of the released compounds (Flynn *et al*., 2008). The composition of DOM released by heterotrophic bacteria is even less known than for phytoplankton, however, they do produce and release organic compounds similar to those released by phytoplankton (Heissenberger and Herndl, 1994; Stoderegger and Herndl, 1998; Carlson, 2002; Kujawinski *et al*., 2009).

Although the largest fraction of DOM remains uncharacterized with respect to molecular identity, phytoplankton exudates consist to a considerable extent of labile compounds such as carbohydrates (mono‐ and polysaccharides), proteins and amino acids (Obernosterer and Herndl, 1995; Myklestad, 2000; Granum *et al*., 2002). Hence, extracellular release of DOM by phytoplankton supports a major fraction of the labile carbon flux in the surface ocean, thereby fueling secondary production (Fogg, 1983; Baines and Pace, 1991; Obernosterer and Herndl, 1995; Morán *et al*., 2013). Labile DOM is defined as being consumed by microbes within hours to days of production but typically only accounts for a fraction of the bacterial C‐ and N‐demand (Fuhrman, 1987). However, the majority of the oceanic DOM pool appears to be recalcitrant with lifetimes from years to millennia, and its sources and sinks are largely unknown (Hansell, 2013).

Novel techniques based on electrospray ionization coupled to mass spectrometry allow for a more detailed characterization of the low molecular weight (LMW, <1000 Da) DOM pool, which represents the major component of DOM and was not analytically accessible before (Kujawinski, 2011). The use of Fourier transform ion cyclotron resonance mass spectrometry (FT‐ICR‐MS) makes it possible to determine elemental formulae from highly accurate mass measurements alone (Kim *et al*., 2016). These recent methodological advances provided new insights into the vast complexity of DOM secreted by phytoplankton and heterotrophic bacteria, suggesting that a major fraction of the released organic molecules is highly diverse and dependent on nutrient availability, the organism itself, and its growth stage (Becker *et al*., 2014; Romano *et al*., 2014; Wienhausen *et al*., 2017; Noriega‐Ortega *et al*., 2019). The DOM composition appears to be highly variable and microbes exude different compounds in the presence of other microbes (Grossart and Simon, 2007). Furthermore, auxotrophy (the inability of an organism to produce a specific compound it requires) and the consequential exchange of a variety of metabolites and precursors between different microbes appear to be more widespread than previously assumed (Morris *et al*., 2011; Carini *et al*., 2014; Garcia *et al*., 2015; Paerl *et al*., 2017).

Ammonia‐oxidizing archaea (AOA) constitute a considerable fraction of microbial biomass in the global ocean, comprising 20%–40% of the ocean's prokaryotic plankton (Karner *et al*., 2001). It is unknown to what extent these chemolithoautotrophic archaea are releasing DOM. If they do, it might be that a significant fraction of marine DOM is of archaeal origin. Furthermore, potential reciprocal interactions via DOM transformations between AOA and heterotrophic bacteria have been suggested previously (Reji *et al*., 2019). Here, we investigated the exometabolomes of three model strains of marine AOA, *Nitrosopumilus adriaticus* NF5, *Nitrosopumilus piranensis* D3C, and *Nitrosopumilus maritimus* SCM1, using untargeted and targeted metabolomics. While untargeted metabolomics aims at detecting and describing as much of the metabolome as possible, targeted metabolomics uses authentic standards to quantify a specific set of compounds (Patti *et al*., 2013). Moreover, we determined the composition of intra‐ and extracellular DFAA in all three strains using high performance liquid chromatography (HPLC).

## Results and discussion

### *Composition of solid phase extracted‐DOM produced by ammonia‐oxidizing archaea*

DOM was extracted from culture supernatant of three strains of AOA, *N. adriaticus* NF5*, N. piranensis* D3C, and *N. maritimus* SCM1. Dissolved organic carbon (DOC) concentrations of solid‐phase extracted DOM (SPE‐DOM) from archaeal cultures harvested during late exponential growth ranged from 3.5 to 5.0 μM, which was on average 2–3 times higher than DOC concentrations of medium blanks (data not shown). We were not able to determine the extraction efficiencies required to remove salts due to the high concentrations of organic HEPES buffer (10 mM) in the culture medium (see Supporting Information), which was not retained by the applied SPE method (see Experimental Procedures) as suggested by the absence of the corresponding mass and molecular formula in our FT‐ICR‐MS analysis. Extraction efficiencies of DOC from seawater are ~60% on average, typically exhibiting a C/N ratio similar to the original DOM (Green *et al*., 2014). However, DOC extraction efficiencies in cultures of heterotrophic bacteria and phytoplankton are typically lower, ranging from 2% to 50% (Becker *et al*., 2014; Lechtenfeld *et al*., 2015; Wienhausen *et al*., 2017). The amount of SPE‐DOC released by the three archaeal strains was significantly lower than has been previously determined for heterotrophic bacteria (~0.04 fmol cell^−1^ compared to ~0.16–1.02 fmol cell^−1^ as in Romano *et al*., 2014). Given the energetic differences between the oxidation of ammonia (ΔG = −271 kJ mol^−1^ NH_3_) and glucose (−2883 kJ mol^−1^ glucose), it is not surprising that these organisms are not releasing as much (fixed) carbon as compared to heterotrophic bacteria. For autotrophic ammonia‐oxidizers, it is much more costly to produce these extracellular compounds. The carbon yield from nitrification of *N. adriaticus* has previously been reported to be ~0.1 (fmol C fixed cell^−1^ d^−1^/ fmol N oxidized cell^−1^ d^−1^) (Bayer *et al*., 2019aa). If we assume an extraction efficiency between 2% and 50%, the fraction of DOC exuded by *Nitrosopumilus* spp. would account for 4%–50% of the fixed carbon in this study.

**Figure 1:**
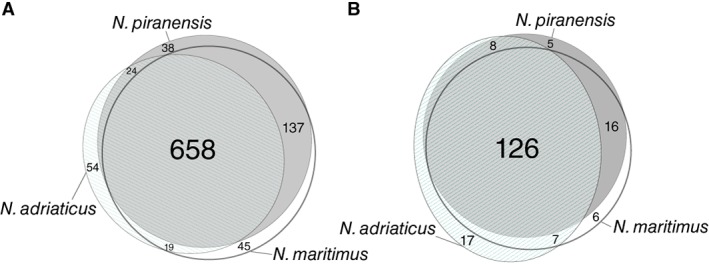
Venn diagram of detected molecular masses (A) and assigned formulae (B) present in at least two technical and two biological replicates per *Nitrosopumilus* strain (see Experimental Procedures).

**Figure 2:**
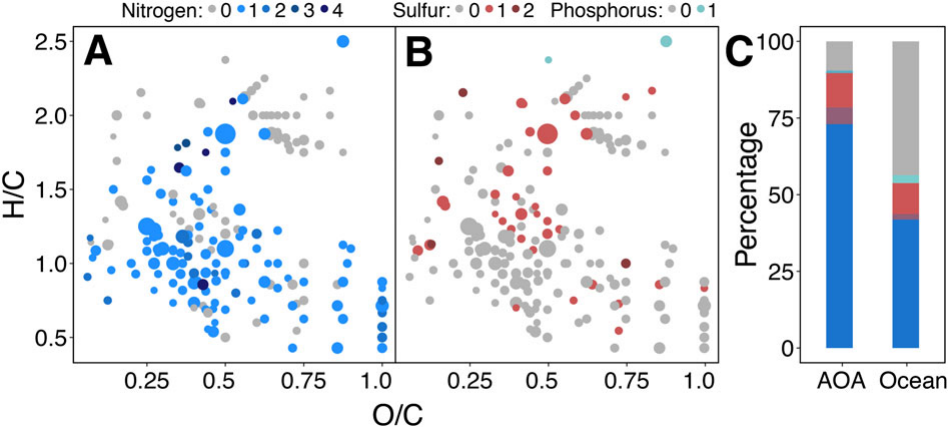
Van Krevelen plots of masses with assigned formulae derived from archaeal exometabolomes containing nitrogen (A), sulfur and phosphorus (B) heteroatoms. Dot sizes correspond to average relative peak intensities. (C) Percentage of assigned formulae in ocean SPE‐DOM and archaeal SPE‐DOM containing nitrogen (blue), sulfur (red) or phosphorus (turquoise) atoms. The presence of two different heteroatoms in one molecular formula is indicated by the overlay of both colours.

A total of 5442 resolved masses of singly charged, intact compounds were detected with FT‐ICR‐MS. After removing masses present in medium controls, 976 masses remained that were present in at least two technical and two biological replicates per strain (see Experimental Procedures for details) (Fig. 1A). Thereof, 185 masses could be assigned to molecular formulae of which 126 were shared by all three strains (Fig. 1B). Of these shared exometabolites, 78.6% contained at least one nitrogen atom, which was much higher than the average percentage of nitrogen‐containing compounds in SPE‐DOM extracted from the North Atlantic (Fig. 2A,C). Previous studies showed that SPE‐DOM released by heterotrophic bacteria is characterized by a high abundance of molecules with heteroatoms (P, N, S) (Romano *et al*., 2014; Lechtenfeld *et al*., 2015). In comparison to the exometabolome of the marine bacterium *Pseudovibrio* sp. FO‐BEG, however, S‐ and P‐containing compounds were much less abundant in the exometabolomes of *Nitrosopumilus* spp. (Romano *et al*., 2014), potentially reflecting the dominance of nitrogen metabolism in AOA (Fig. 2B,C).

**Figure 3:**
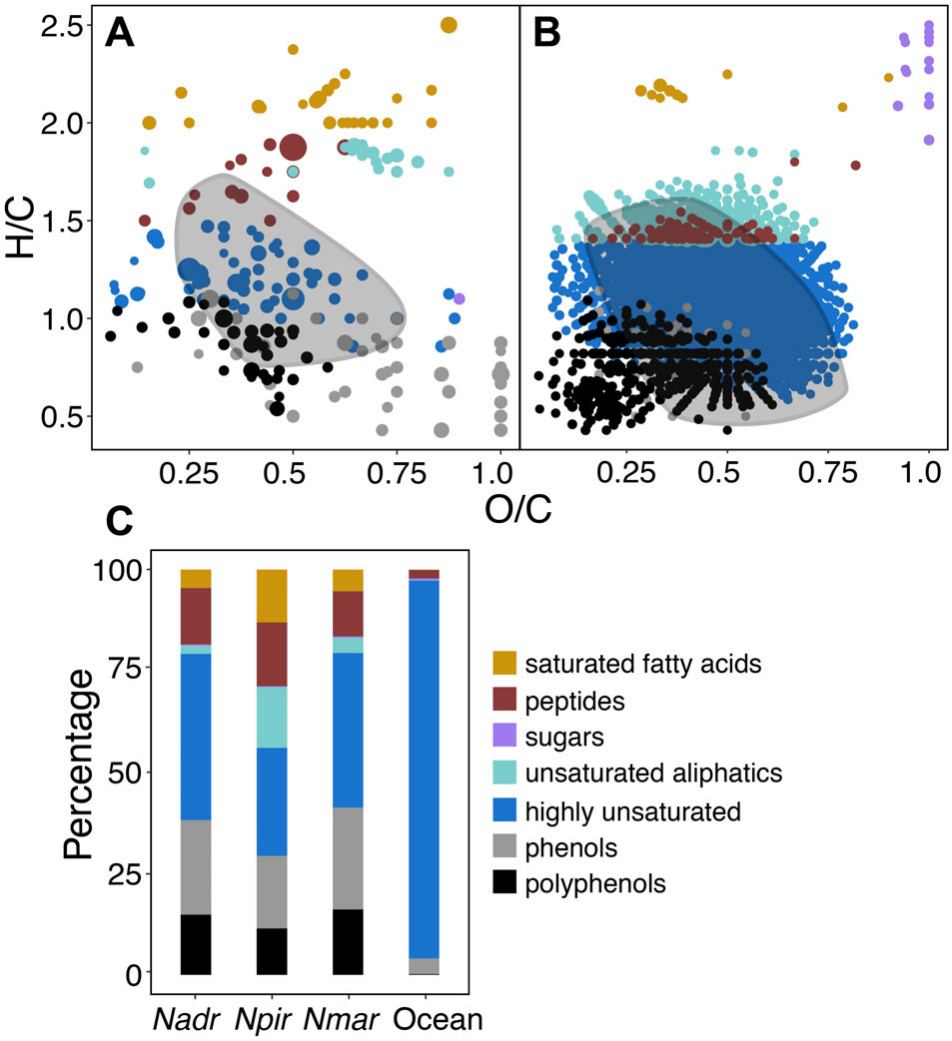
Categorization of assigned formulae obtained from archaeal SPE‐DOM (A) and ocean SPE‐DOM (B) into compound classes (for definition of categories see Experimental Procedures). Dot sizes correspond to average relative peak intensities and carboxyl‐rich alicyclic molecules (CRAM) are indicated by grey clouds. (C) Comparison of the SPE‐DOM compositions of three *Nitrosopumilus* strains (*N. adriaticus* NF5*, N. piranensis* D3C*, N. maritimus* SCM1) with ocean SPE‐DOM signatures normalized to relative peak intensities.

**Figure 4:**
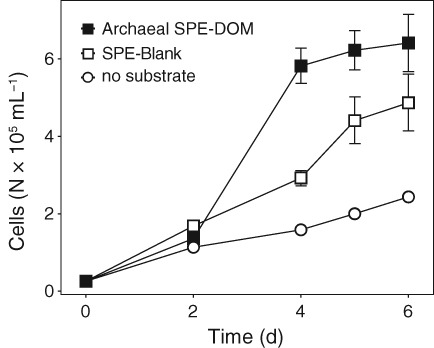
Growth of the heterotrophic alphaproteobacterium *O. alexandrii* on SPE‐DOM derived from *Nitrosopumilus* cultures (black squares) and culture medium (white squares) in comparison to control incubations without addition of organic carbon (white circles). Error bars represent standard deviations of measurements from triplicate cultures.

The number of detected masses with assigned molecular formulae was much lower in SPE‐DOM of *Nitrosopumilus* spp. than observed for heterotrophic bacteria using comparable methodology (Romano *et al*., 2014; Wienhausen *et al*., 2017). However, it remains unclear whether the exometabolomes of chemolithoautotrophic AOA are generally less diverse as compared to heterotrophic bacteria, or whether *Nitrosopumilus* spp. produce fewer compounds that are retained by SPE columns. SPE‐DOM is primarily concentrated based on polarity when using PPL cartridges (Dittmar *et al*., 2008), and is relatively rich in low‐molecular weight compounds and carboxyl‐rich alicyclic molecules (CRAM). CRAM accounted for 31% of all archaeal SPE‐DOM molecules (Fig. 3A), which is comparable to previously reported CRAM production by bacterioplankton (Lechtenfeld *et al*., 2015). It has been suggested that CRAM are largely comprised of decomposition products of biomolecules, representing a major refractory component of oceanic DOM (Hertkorn *et al*., 2006). However, the rapid generation of CRAM by archaeal and bacterial metabolism (Lechtenfeld *et al*., 2015; this study) challenges their potentially refractory nature. If such a large proportion of freshly produced DOM was refractory, then the oceans would be filled almost exclusively by CRAM. Furthermore, recent evidence suggests that the low concentrations of individual compounds rather than their inherent recalcitrance might prevent prokaryotic consumption of a substantial fraction of DOM in the ocean (Arrieta *et al*., 2015). In contrast, Shen and Benner (2018) showed in bioassay experiments that C‐18 extracted DOC appears to be refractory and further utilization of these molecules might require favourable environmental conditions (e.g. photodegradation). Nevertheless, SPE‐DOM from *Nitrosopumilus* spp. supported the growth of a heterotrophic alphaproteobacterium (Fig. 4), suggesting that at least a fraction of the released DOM retained on SPE columns might be used as substrate by heterotrophic bacteria.

While oceanic SPE‐DOM is dominated by highly unsaturated compounds, *Nitrosopumilus*‐derived SPE‐DOM contained a high proportion of compounds with molecular formulae characteristic for phenols, polyphenols, highly unsaturated compounds, unsaturated aliphatics, peptides and saturated fatty acids (Fig. 3A,C). The exometabolomes of AOA contained relatively fewer sugars and peptides, and a higher percentage of phenols compared to *Pseudovibrio* sp. FO‐BEG (Romano *et al*., 2014). The production of phenols and polyphenols has been associated with antioxidant activity, suggested to be caused by increased oxidative stress (Romano *et al*., 2014). *Nitrosopumilus* spp. produce high amounts of the oxidant H_2_O_2_ as a result of their metabolic activity, which ultimately inhibits their growth (Kim *et al*., 2016; Bayer *et al*., 2019bb). Although, the H_2_O_2_‐detoxifying enzyme catalase was initially added to the culture medium, catalase activity gradually decreases with time, potentially increasing the exposure to oxidative stress in *Nitrosopumilus* cultures. A relatively high proportion of the assigned molecular formulae of the exometabolomes of all three strains had elemental ratios characteristic for saturated fatty acids (Fig. 3A,C). In contrast to bacteria, archaeal membrane lipids consist of isoprenoids and do not contain fatty acids (Koga and Morii, 2007). However, fatty acids have been detected in several Crenarchaeota species and in halorhodopsins of *Haloarchaea* (Kolbe *et al*., 2000; Hamerly *et al*., 2015). Recently, it was suggested that Archaea potentially synthesize fatty acids by reversing the β‐oxidation pathway (Dibrova *et al*., 2014). *Nitrosopumilus* spp. genomes encode putative enzymes involved in this proposed pathway, including enoyl‐CoA hydratase, 3‐hydroxyacyl‐CoA dehydrogenase, and acetyl‐CoA C‐acetyltransferases, as well as for a distant homologue of FadE that could potentially exhibit acyl‐CoA dehydrogenase activity (Dibrova *et al*., 2014). Nevertheless, as compound classes are assigned based on elemental ratios without structural information (see Experimental Procedures), highly saturated molecules with oxygen‐containing functional groups other than carboxylic acids could fall within the same compound class.

### *Release of ecologically relevant metabolites by three* Nitrosopumilus *strains*

Targeted exometabolomics revealed that only a small fraction of the 92 specific compounds tested was identified in SPE‐DOM of the three *Nitrosopumilus* strains. (Table 1). A major feature observed in the targeted analysis of the extracellular metabolite fractions was the presence of the nucleoside thymidine, which was the most abundant identified metabolite in all biological replicates of each strain, ranging between 3.64 and 15.07 nM when corrected for extraction efficiency (Table 1). The amount of thymidine in SPE‐DOM would represent ~10% of the total cellular thymidine content contained in DNA in the cultures (calculated based on the dsDNA weight of 1 fg Mb^−1^ and a G + C content of 34 mol%). However, cultures were harvested in late exponential growth phase and no decrease in cell abundance was observed (data not shown), suggesting that thymidine was released by growing cells. Thymidine has also been reported as a dominant molecule in the exometabolome of the cyanobacterium *Synechococcus elongatus* (Fiore *et al*., 2015). Like *Synechococcus*, the genomes of members of the *Nitrosopumilus* genus lack thymidine kinase, which phosphorylates thymidine to thymidine monophosphate (TMP) in the thymidine salvage pathway. Instead, uridine 5′‐monophosphate (UMP) is converted to TMP by thymidylate synthase for DNA synthesis. Thus, thymidine released by TMP hydrolysis or DNA degradation cannot be metabolized and is likely excreted as waste product (Fiore *et al*., 2015). Thymidine represents a valuable substrate for heterotrophic bacterioplankton and is readily taken up and incorporated into DNA, a feature that has been applied to measure heterotrophic prokaryotic production in aquatic environments (Fuhrman and Azam, 1982; Chin‐Leo and Kirchman, 1988).

**Table 1:** Targeted exometabolomics of *Nitrosopumilus*‐derived SPE‐DOM.

Metabolite	*N. piranensis* D3C	*N. adriaticus* NF5	*N. maritimus* SCM1	Extraction efficiency (%)
Thymidine	3.64–15.07	4.46–9.41	4.81–12.70	51.1 ± 14.3
Pantothenic acid	0.12–0.18	0.22–0.54	0.16–0.24	50.1 ± 8.5
Riboflavin^a^	0.10‐0.15	0.09‐0.15	0.09–0.10	106.1 ± 4.0
Adenine	d.	d.	d.	n.r.
N‐acetylglucosamine	d.	n.d.	n.d.	n.r.
Proline	d.	d.	d.	n.r.
Isethionic acid	d.	d.	d.	n.r.

Metabolite concentrations are given in nM and represent the minimum and maximum values detected in three biological replicates per strain. The presented values have been corrected for extraction efficiencies in Milli‐Q water as determined by Johnson *et al*. (2017), and the complete list of metabolites can be found in Kido Soule *et al*. (2015). d., detected; n.d., not detected; n.r., not retained.

a Riboflavin was detected in one out of two blanks at 0.04 nM.

Minor components of the exometabolomes might be ecologically important even at low concentrations, such as pantothenic acid (vitamin B_5_) and riboflavin (vitamin B_2_), which were found at concentrations of 120–540 pM and 90–150 pM, respectively, in all three cultures (Table 1). B vitamins are some of the most commonly required biochemical cofactors for cellular metabolism, yet their concentrations vary from undetectable to a few picomoles per litre in ocean waters (Menzel and Spaeth, 1962; Sanudo‐Wilhelmy *et al*., 2012). Riboflavin is the key precursor of flavin mononucleotide (FMN) and flavin adenine dinucleotide (FAD), cofactors required for flavoprotein‐mediated cellular redox reactions (García‐Angulo, 2017). Furthermore, riboflavin has been shown to be involved in extracellular processes, including extracellular electron transfer and quorum sensing signalling (Rajamani *et al*., 2008; García‐Angulo, 2017). Pantothenic acid is used for the synthesis of coenzyme A (CoA), an essential cofactor in the reaction mechanism of the tricarboxylic acid cycle (Leonardi and Jackowski, 2007) and the carbon fixation pathway of AOA, the 3‐hydroxypropionate/4‐hydroxybutyrate cycle (Könneke *et al*., 2014).

Adenine, proline and isethionic acid were detected in the exometabolomes of all three strains, yet not in every biological replicate (Table 1). This is probably due to the fact that these compounds are typically not retained by extraction with PPL, albeit potentially present in high amounts in our study. Furthermore, the amino sugar *N*‐acetylglucosamine was only detected in SPE‐DOM of *N. piranensis* D3C, yet in all three biological replicates (Table 1), indicating that *N. piranensis* D3C might produce higher amounts of this metabolite as compared to the other two strains. While Archaea lack peptidoglycan, which largely consists of *N*‐acetylglucosamine and *N*‐acetylmuramic acid, archaeal S‐layers are often heavily glycosylated and glycan composition has been shown to vary even between very closely related archaeal species (Albers and Meyer, 2011).

**Figure 5:**
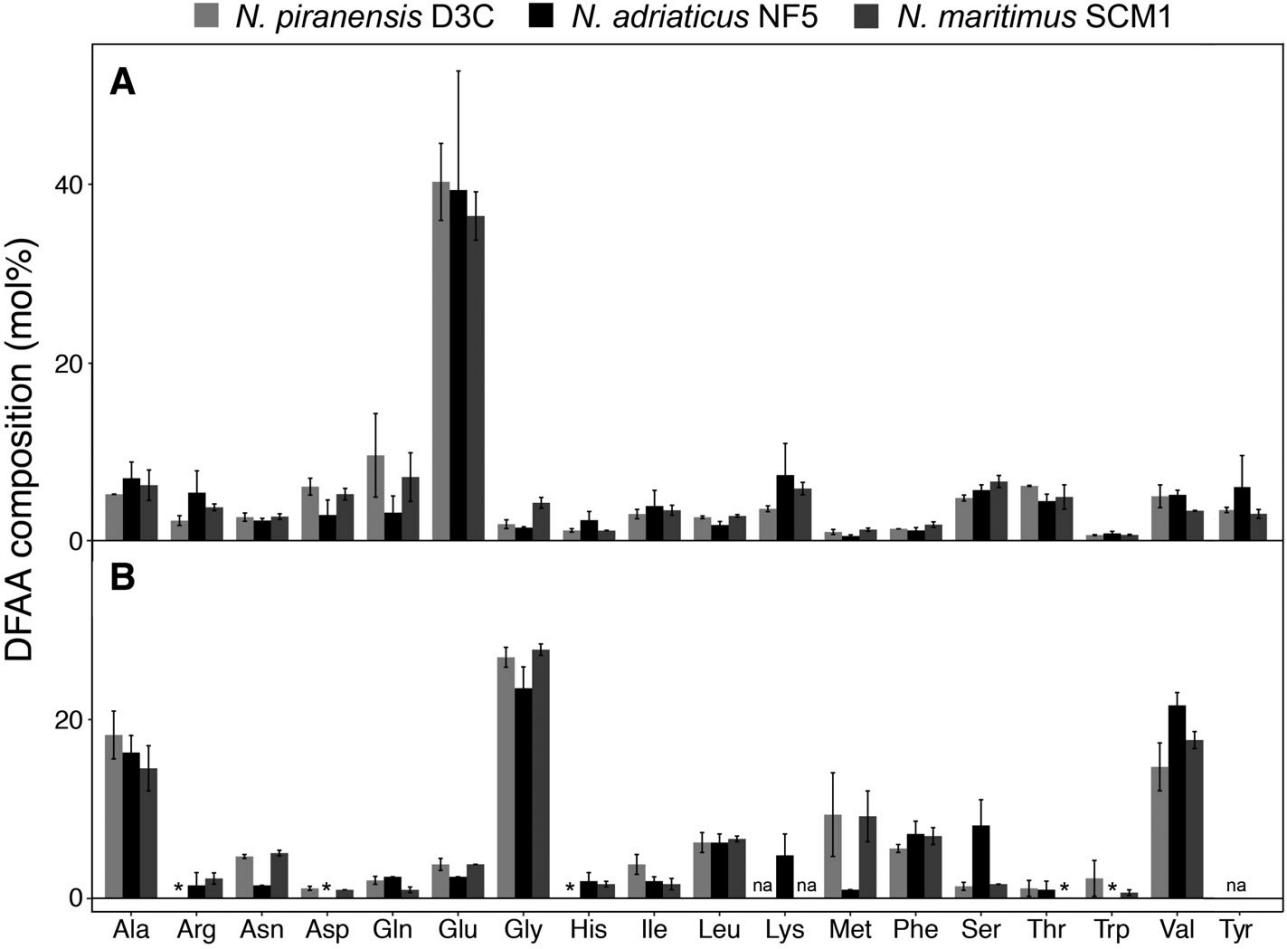
Intracellular (A) and extracellular (B) dissolved free amino acid (DFAA) composition of *N. piranensis* D3C, *N. adriaticus* NF5, and *N. maritimus* SCM1. Error bars represent standard deviations of measurements from triplicate cultures. *, values below detection limit; na, not available.

We also determined concentrations of DFAA in the culture medium throughout the growth of all three *Nitrosopumilus* strains. Total DFAA concentrations ranged between 261 and 454 nM at the end of exponential growth, and net cell‐specific DFAA release rates were between 2.6 and 16.4 amol cell^−1^ d^−1^ (with *N. piranensis* exhibiting the highest rates) (Table 2), which is two to three orders of magnitude lower as compared to rates previously reported for marine phytoplankton species (0.17–38 fmol cell^−1^ d^−1^) (Martin‐Jézéquel *et al*., 1988; Myklestad *et al*., 1989). Surprisingly, *Nitrosopumilus* spp. released DFAA at higher rates than two heterotrophic alphaproteobacterial strains of the globally abundant *Roseobacter* clade (concentrations were below the detection limit of 0.5 nM) (Wienhausen *et al*., 2017). Furthermore, the extracellular DFAA composition appeared to be, at least to some extent, species‐specific as shown for different phytoplankton species (Sarmento *et al*., 2013). *Nitrosopumilus adriaticus* NF5 released higher concentrations of serine, whereas *N. piranensis* D3C and *N. maritimus* SCM1 released higher concentrations of methionine and asparagine (Fig. 5A, Supporting Information Table S3). When exposed to oxidative stress, cell‐specific DFAA release rates increased, in particular those of glutamine and threonine, as compared to cultures grown under non‐stress conditions (Supporting Information Fig. S2).

**Table 2:** Extracellular total dissolved free amino acid (DFAA) concentrations produced by the three *Nitrosopumilus* strains.

Strain	Time	Cells	Total DFAA	+Val, Met	Release rates^a^
	(days)	(x10^9^ L^−1^)	(nM)	(nM)	(amol cell^−1^ d^−1^)
NF5	0	3.1			
NF5	3	10.7	26 (17)		3.4 (2.2)
NF5	5	23.5	135 (24)		8.5 (0.5)
NF5	7	31.1	164 (33)	209 (36)	3.8 (1.2)
NF5	9	34.6	214 (44)	261 (40)	14.3 (1.1)
D3C	0	2.1			
D3C	3	5	44 (42)		15.2 (14.5)
D3C	6	11.7	154 (27)		16.4 (2.2)
D3C	9	29.4	346 (51)	454 (84)	9.0 (4.5)
SCM1	0	1.8			
SCM1	3	7.2	14 (14)		2.6 (2.6)
SCM1	6	29.4	164 (63)	239 (73)	6.8 (2.2)
SCM1	8	48.2	235 (22)	320 (34)	3.8 (2.2)

Standard deviations of measurements from triplicate cultures are shown in parentheses.

a Valine and methionine were excluded from per‐cell DFAA release rates as they could only be quantified in the absence of NH_4_^+^/NH_3_, corresponding to the end of exponential growth (see Experimental Procedures).

### *Potential mechanisms of DFAA release by ammonia‐oxidizing archaea*

The fraction of DOM released by phytoplankton is highly variable, accounting for up to 62% of the total photosynthetically fixed carbon (Carlson, 2002). Phytoplankton exudation has largely been interpreted as the active release of excess photosynthates when carbon fixation exceeds incorporation into cell material (Fogg, 1983). However, Bjørrisen (1988) speculated already more than 30 years ago that diffusion rather than overflow metabolism might be the responsible mechanism of the photosynthetic extracellular release.

Among the three *Nitrosopumilus* strains, the intracellular DFAA composition was highly similar, with glutamic acid dominating the intracellular DFAA pool (35–50 mol%) (Fig. 5A). With the exception of glutamic acid, the intracellular DFAA composition largely reflected the average amino acid composition encoded by *Nitrosopumilus* genomes (Supporting Information Table S4). Ammonia is typically incorporated into biomolecules through glutamate and thus, glutamate/glutamic acid is present at elevated concentrations in most cells, and has been shown to dominate the intracellular metabolome of *Escherichia coli* (Bennett *et al*., 2009). Furthermore, various bacteria and archaea accumulate glutamate in their cells to counteract external osmotic pressure (Roberts, 2005). Intriguingly, glutamic acid was largely absent from the extracellular DFAA pool of exponentially growing cells (Fig. 5B). Instead, hydrophobic amino acids, including alanine, glycine, valine, leucine, isoleucine, phenylalanine, methionine, and tryptophan made up ~80% of the extracellular DFAA of the three *Nitrosopumilus* strains during late exponential growth phase. Additionally, proline, which is also highly hydrophobic, was detected via targeted exometabolomics (Table 1). The permeability of the lipid bilayer for hydrophobic amino acids might be up to 100 times higher as compared to hydrophilic amino acids, as indicated in experiments with artificial lipid vesicles (Chakrabarti and Deamer, 1992). Furthermore, amino acid transport mutants of the cyanobacterium *Anabaena* were shown to release a mixture of hydrophobic amino acids, supporting the hypothesis of passive diffusion (Pernil *et al*., 2015). We did not identify putative amino acid transporters in the proteomes of either strain (Bayer *et al*., *unpublished*), suggesting that the extracellular DFAA composition is determined by selective release rather than selective re‐uptake of amino acids by the cells.

**Figure 6:**
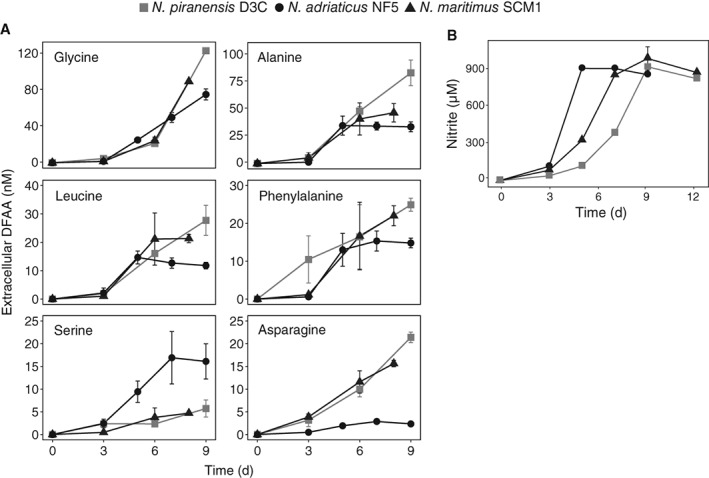
Net release of dissolved free amino acids (DFAA) (A) and nitrite production (B) throughout the growth of three *Nitrosopumilus* strains. T0 concentrations were subtracted from all time points (see Experimental Procedures). Error bars represent standard deviations of measurements from triplicate cultures. Note that the scale bars are different between different amino acid species.

The release of most amino acids followed the growth patterns of each strain, as indicated by nitrite production and cell abundance measurements (Fig. 6, Supporting Information Table S3), with the exception of glycine which was continuously released at high rates. The cleavage of L‐serine to glycine results in the formation of 5,10‐methylenetetrahydrofolate (from tetrahydrofolate), which represents an important one‐carbon unit donor for pyrimidine, purine and methionine biosynthesis (Schirch *et al*., 1985). The decoupling of glycine release (and its precedent production) from ammonia oxidation might indicate that pyrimidine/purine biosynthesis is maintained at a similar level by the cell even when reaching stationary phase.

### *DOM release by AOA: implications for the microbial food web*

The inability of an organism to synthesize a particular compound required for its growth (auxotrophy) appears to be more widespread than previously assumed (Millette *et al*., 2018). Exploiting a resource produced by another organism rather than *de novo* synthesizing a specific compound has been suggested to provide a selective advantage leading to reductive genome evolution (‘Black Queen Hypothesis’) (Morris *et al*., 2012). The auxotrophy for B vitamins in phytoplankton has been known for decades (Droop, 1957). However, a recent survey of bacterial genomes (from various environments) revealed that only 1% encoded the complete pathways for all 20 essential amino acids (Mee and Wang, 2012). The heterotrophic marine bacterium *Pelagibacter ubique*, a member of the SAR11 clade, known as the most abundant organisms on the planet (Morris *et al*., 2002), lacks biosynthetic pathways for various vitamins (B_1_, B_5_, B_6_, B_7_, B_12_) and requires pyruvate, glycine, and organic sulfur (e.g., methionine) for growth (Giovannoni *et al*., 2005; Tripp *et al*., 2008; Carini *et al*., 2013). Recently, it was shown in co‐culture experiments that photoautotrophic *Prochlorococcus* strains can fulfil some of the unique metabolic requirements of SAR11 (Becker *et al*., 2019). Additionally, members of the *Roseobacter* group have been suggested as important suppliers of so‐called public goods by releasing growth factors as well as biosynthetic precursors, including various vitamin B precursors (Wienhausen *et al*., 2017). AOA are prototrophic for many B vitamins and have previously been suggested to play a critical role for the microbial metabolic activity in meso‐ and bathypelagic waters by supplying these essential nutrients (Santoro *et al*., 2015; Heal *et al*., 2017). Our results provide the first evidence for the release of B vitamins by marine AOA. Curiously, concentrations of multiple B vitamins were shown to be enriched primarily in the upper mesopelagic zone (Sanudo‐Wilhelmy *et al*., 2012) where AOA typically exhibit peaks of abundance and activity (Santoro *et al*., 2019).

Phytoplankton are considered the major producers of DOM in the surface ocean, accounting for a large fraction of labile carbon flux which fuels heterotrophic production (Fogg, 1983; Baines and Pace, 1991). Below the sunlit surface layers of the ocean, chemolithoautotrophy is a widespread strategy and this ‘dark primary production’ has a major impact on global carbon cycling (Reinthaler *et al*., 2010). Our data suggest that AOA might release a fraction of the fixed carbon into the ambient water in the form of amino acids and other metabolites, which has not been quantified thus far. Quantifying the chemolithoautotrophic contribution to the DOM pool and its turnover might represent a potentially important food source for deep ocean microbes. The average prokaryotic heterotrophic carbon demand in the deep North Atlantic amounts to ~20 μmol C m^−3^ d^−1^, as estimated from mean heterotrophic prokaryotic production (~2.8 μmol C m^−3^ d^−1^) (Herndl *et al*., 2005; Reinthaler *et al*., 2006) and an average growth efficiency of 14% (Carlson and Ducklow, 1996). If we assume that AOA in their natural environment release a similar fraction (4%–50%) of fixed carbon (~0.42 μmol C m^−3^ d^−1^ (Herndl *et al*., 2005; Varela *et al*., 2011)) as in our lab experiments, the DOC released by AOA would account only for 0.08%–1.05% (of which ~0.003% are released as DFAA) of the heterotrophic prokaryotic carbon demand in the deep North Atlantic.

## Conclusions

This study characterizes the exometabolome of autotrophic AOA. Untargeted and targeted exometabolomic profiling of DOM from three model strains of AOA suggest that these abundant chemolithoautotrophs might influence the chemical composition of the oceanic DOM. Our results provide evidence that AOA release a suite of organic compounds with potentially varying reactivities. However, the rapid generation of CRAM by AOA challenges the paradigm that CRAM are solely representative for old and refractory compounds in the global ocean.

The release of fixed carbon represents an unprecedented link between chemolithoautotrophic production and heterotrophic consumption of DOM in the ocean and has potentially important implications for the role of AOA in the microbial carbon pump. The potential contribution of the released DOC to fuel heterotrophic activity represents ~0.08%–1.05% of the heterotrophic prokaryotic carbon demand in the deep North Atlantic. Despite this overall low contribution to the prokaryotic carbon demand, the release of ecologically and biochemically relevant metabolites could potentially be crucial for microbes that are auxotrophic for some of these compounds, including members of the globally abundant and ubiquitous SAR11 clade.

## Experimental procedures

### *Culture conditions*

Axenic cultures of *N. adriaticus* NF5, *N. piranensis* D3C, and *N. maritimus* SCM1 were grown in Synthetic Crenarchaeota Medium (SCM) as previously described (Martens‐Habbena *et al*., 2009; Bayer *et al*., 2016, also see Supporting Information), with addition of 5 units mL^−1^ catalase (Bayer *et al*., 2019b). All plastic and glassware used were rinsed with acidified ultrapure water (Milli‐Q, HCl analytical grade, pH 2), and glassware was combusted at 500°C for 5 h. Cultures were grown in 2 l glass bottles for targeted and untargeted exometabolomics analysis and in 30 ml polypropylene tubes (Sterilin, Thermo Fisher Scientific) for DFAA analysis. For exometabolomics, cultures were grown until reaching mid‐exponential growth phase (after approx. 7 days), yielding cell abundances of ~5 × 10^7^ cells ml^−1^. Growth was monitored by measuring nitrite production and cell abundance as previously described (Bayer *et al*., 2016).

### *Untargeted and targeted exometabolomics*

Culture supernatants of 1–2 l of culture were obtained via centrifugation (14,000x *g*, 10°C, for 1 h) and subsequent filtration twice through 0.2 μm polycarbonate filters (GTTP, Millipore). DOM was extracted from culture supernatants by solid phase extraction (SPE) as previously described (Dittmar *et al*., 2008). Briefly, supernatants were acidified to pH 2 (HCl, analytical grade) and extracted on Bond Elut PPL sorbent cartridges (1 g, Agilent, Waldbronn, Germany). Subsequently, cartridges were rinsed with 2 × 6 ml acidified (pH 2) ultrapure water, air‐dried, eluted with 6 ml methanol into amber glass vials, and stored at −20°C. Procedural blanks were prepared by processing culture medium in the same way. Three biological replicates per archaeal strain were extracted. Due to the high culture volumes required, biological replicates were performed consecutively and culture medium blanks were generated for every medium batch (Supporting Information Fig. S1). DOC concentrations of the solid‐phase extracted DOM were quantified as previously described (Osterholz *et al*., 2014). Briefly, aliquots of methanol extracts were evaporated and re‐dissolved in acidified (pH 2) ultrapure water. Samples were subsequently analysed by high temperature catalytic combustion using a Shimadzu TOC‐VCPH/CPN Total Organic Carbon Analyzer (Shimadzu) and DOC concentrations were calculated by taking into account the concentration factor of the SPE‐DOM extracts.

Untargeted mass spectrometric analysis of DOM extracts was performed on a solariX Fourier transform ion cyclotron resonance mass spectrometer with a 15 Tesla magnet (Bruker, Daltonics, Bremen, Germany). The system was equipped with an electrospray ionization source (ESI, Bruker Apollo II, Daltonics, Bremen, Germany) applied in negative ionization mode. Prior to injection, methanol extracts were mixed with ultrapure water (50:50 v/v) and subsequently diluted with methanol: ultrapure water (50:50 v/v) according to the extracted volume (1:1 for 1 l, 1:2 for 2 l). The injected concentrations ranged between 5 and 7 ppm. For each measurement, 300 scans were accumulated in the mass window of 92 to 1000 Da. Spectra were calibrated internally with a reference mass list using the Bruker Daltonic Data Analysis software Version 4.0 SP4. Instrument performance was verified using an in‐house reference sample of North Equatorial Pacific Intermediate Water (NEqPIW) as previously described (Osterholz *et al*., 2014; Riedel and Dittmar, 2014). Detected mass to charge (*m/z*) ratios were processed by applying a customized routine Matlab script and molecular formulae were assigned using the elements C, H, O, N, S and P according to the guidelines established previously (Koch *et al*., 2007). Masses detected in only one sample were removed as well as those with a maximum signal‐to‐noise ratio < 4 across all samples. Technical triplicates were measured for every biological replicate and only peaks detected in at least two technical and two biological replicates were kept in the dataset. We chose this less stringent filtering approach to include low‐intensity peaks but observed the same general trends in chemical composition when only including masses present in all technical and biological replicates (data not shown). Peaks of procedural blanks were removed from all archaeal exometabolome data if they were present in more than half of all blank replicates (technical + biological). Peaks of procedural blanks present in less than half of all blank replicates were only kept in the dataset if average peak intensities were at least two times lower than in archaeal exometabolome samples. Data were normalized to the sum of all peak intensities and assigned formulae were categorized into compound classes as described in Romano *et al*. (2014): peptides (H/C = 1.5–2.0, O/C < 0.9, contains N), sugars (O/C ≥ 0.9, AI_mod_ < 0.5), saturated fatty acids (H/C ≥ 2, O/C ≤ 0.9), unsaturated aliphatic compounds (H/C = 1.5–2.0, O/C < 0.9, does not contain N), highly unsaturated compounds (AI_mod_ < 0.5, H/C < 1.5, O/C < 0.9), phenols (AI_mod_ ≥ 0.5, less than 12 C atoms), and polyphenols (AI_mod_ ≥ 0.5, more than 12 C atoms). CRAMs were identified as previously reported by Hertkorn *et al*. (2006): DBE/C (0.30–0.68), DBE/H (0.20–0.95) and DBE/O (0.77–1.75), where the molecular double bond equivalent (DBE) was calculated as DBE = 1 + ½(2C‐H + N + P). The raw data and the filtered dataset can be found in the Supporting Information. The exometabolome of the three archaeal strains obtained by FT‐ICR‐MS was compared with the SPE‐DOM from the water column of the North Atlantic acquired and analysed with the same approach (Hansman *et al*., 2015). Area‐proportional Venn diagrams were constructed with the program eulerAPE (Micallef and Rodgers, 2014).

For targeted analysis, DOM extracts were dried down and re‐dissolved in 95:5 (v/v) water:acetonitrile with deuterated biotin (final concentration 0.05 mg mL^−1^) which served as an injection standard. Samples were then analyed by ultra‐high performance liquid chromatography (Accela Open Autosampler and Accela 1250 Pump, Thermo Scientific) coupled to a heated electrospray ionization source (H‐ESI) and a triple quadrupole mass spectrometer (TSQ Vantage, Thermo Scientific) operated under selected reaction monitoring (SRM) mode. Chromatographic separation was performed on a Waters Acquity HSS T3 column (2.1 × 100 mm, 1.8 μm) equipped with a Vanguard pre‐column and maintained at 40°C. The column was eluted with (A) 0.1% formic acid in water and (B) 0.1% formic acid in acetonitrile at a flow rate of 0.5 ml min^−1^ as previously described (Kido Soule *et al*., 2015). Separate autosampler injections of 5 μl each were made for positive and negative ion modes. The samples were analysed in a random order with a pooled sample run after every six samples. The mass spectrometer was operated in SRM mode. Optimal SRM parameters (s‐lens, collision energy) for each target compound were optimized individually using an authentic standard as previously described (Kido Soule *et al*., 2015). Two SRM transitions per compound were monitored for quantification and confirmation. Eight‐point external calibration curves based on peak area were generated for each compound. The resulting data were converted to mzML files using the msConvert tool (Chambers *et al*., 2012) and processed with MAVEN (Melamud *et al*., 2010). Compounds were considered when they were present in at least two out of three biological replicates and absent in media blanks. It should be noted that due to sample limitations, it was not possible to generate an internal calibration curve. Hence, we could not account for potential matrix effects in this study and thus, the concentrations of metabolites quantified with targeted metabolomics might be over‐ or underestimated.

### *Growth of the alphaproteobacterium Oceanicaulis alexandrii on Nitrosopumilus‐derived SPE‐DOM*.

*Oceanicaulis alexandrii* was the last contaminant of *N. adriaticus* NF5 and *N. piranensis* D3C enrichment cultures prior to their isolation into pure culture, and has previously been shown to be able to grow in co‐culture with all three investigated *Nitrosopumilus* strains (Bayer *et al*., 2019b). In this study, the alphaproteobacterium was grown axenically in SCM medium amended with 100 μM amino acids (alanine, aspartic acid, glutamic acid, serine) in the dark at 30°C without shaking, and growth was monitored by flow cytometry (Marie *et al*., 1999). After reaching late exponential growth, *O. alexandrii* was starved for 3 days and then transferred into nine 20 ml flasks containing fresh SCM medium (1% inoculum). To three replicates, *Nitrosopumilus‐*derived SPE‐DOM and SPE‐DOM from culture medium controls was added, respectively, and three replicates served as no substrate control incubations. Methanol extracts (1.2 ml each) were dried down in a SpeedVac (Eppendorf, Concentrator Plus), subsequently re‐dissolved in SCM medium and sterile‐filtered through 0.1 μm syringe filters (Millipore Durapore, 25 mm). DOM extracts were added to 20 ml of *O. alexandrii* culture, resulting in a 20X concentration as compared to the initial DOC concentrations retained on SPE columns (approx. 40 μM of *Nitrosopumilus*‐derived DOM and 20 μM of medium blank SPE‐DOM).

### *Determination of dissolved free amino acids*

For the analysis of extracellular DFAA, 1 ml of culture was filtered through 0.2 μm syringe filters with polyvinylidene difluoride (PVDF) membrane (Whatman® Puradisc 13) and the filtrate was stored in 1.5 ml combusted amber glass vials at −20°C until analysis. For the analysis of intracellular DFAA, cells were harvested via centrifugation as described above and 1.5 ml Milli‐Q was added to the cell pellets. Cells were subsequently lysed with 4 freeze/thaw cycles and the lysate was filtered through 0.1 μm syringe filters (Millipore Durapore, 25 mm). Concentrations of extracellular DFAA were determined throughout the growth of the three strains and intracellular DFAA were determined at late exponential growth phase in all three strains.

Intra‐ and extracellular DFAA were analysed using a HPLC system (Agilent 1260 Infinity Bioinert) equipped with a Zorbax Sq‐Aq Analytical guard column (4.6 x 12.5 mm, 5 μM) and Zorbax Eclipse AAA Rapid resolution column (4.6 x 150 mm, 3.5 μM; Agilent Technologies, Santa Clara). To 1 ml of sample, 75 μl borate buffer (0.4 M, pH 10.2) and 5 μl OPA reagent (5061–3335, Agilent Technologies) were added for the derivatization reaction (27°C, 2 min). The injection volume was 100 μl and separation was achieved by applying a flow rate of 0.8 ml min^−1^ under a constant column temperature of 25°C using a modified gradient to reduce interference of high ammonium concentrations (Supporting Information Table S1) (Taubner *et al*., *in preparation*). Excitation and emission wavelengths of the fluorescence detector were 340 nm and 450 nm, respectively, with gain factor 12 for quantification. For peak identification and area calculation, a primary amino acid standard (A2161, Sigma‐Aldrich) was prepared in the range of 1 nM to 1 μM. Spectra were analysed with the software Agilent ChemStation (Agilent Technologies).

Limits of detection (LOD) and limits of quantification (LOQ), as well as the recovery of individual amino acids in Milli‐Q water and culture medium can be found in the Supporting Information. Extracellular methionine and valine concentrations could not be reliably quantified until late exponential/early stationary growth phase of the cultures due to the interference with ammonium in the culture medium. Tyrosine could never be reliably quantified in extracellular samples due to ammonium interference and the presence of three peaks with similar retention times. Lysine could only be measured in the culture supernatant of *N. adriaticus* NF5, due to other compound(s) with similar retention times present at micromolar concentrations in the medium of *N. piranensis* D3C and *N. maritimus* SCM1. Aspartic acid, serine, glutamine and histidine often appeared as small double peaks, which were integrated together for consistency.

## Supporting information


**Appendix S1**: Supplementary Information

**Appendix S2**: Supplementary Files
