# Supplementary material for: Ammonia‐oxidizing archaea release a suite of organic compounds potentially fueling prokaryotic heterotrophy in the ocean
Source: Environ Microbiol. 2019 Aug 6;21(11):4062–75. doi: 10.1111/1462-2920.14755 (PMC6899801; doi:10.1111/1462-2920.14755)
Supplement: Supplementary file 1 — Appendix S1: Supplementary Information [file EMI-21-4062-s001.docx]

Supplementary Information (SI)

**SI Methods**

**Principal coordinates analysis of archaeal SPE-DOM**

Principal coordinates analysis (PCoA, = Multidimensional scaling, MDS) of all resolved masses of compounds detected in all three *Nitrosopumilus* strains and medium blanks was performed in R (version 3.5.0) (R Core Team, 2013). Bray-Curtis distance matrices were calculated using the normalized peak intensities and analysis was performed using the vegan package (version 2.5-3) (Oksanen *et al.*, 2013).

**﻿Accuracy and precision of dissolved free amino acid (DFAA) determination with HPLC**

Spent culture medium (containing µM ammonium concentrations) and Milli-Q test samples were amended with three concentrations of amino acid standard mix (5 nM, 50 nM, 500 nM). Five replicates for each concentration were measured. Recovery of DFAA in Milli-Q and culture medium was determined by calculating the average recovery from amino acid standards (Table S2).

The recovery R (in %) was estimated as:

$$R=\frac{(Ca-Cu)}{Cs\times100}$$

where Ca and Cu are the concentrations of the amino acids in amended and unamended samples, respectively, and Cs is the concentration of the amino acid standard added to the test samples.

*R* ranged from 92.1% to 115.4% in Milli-Q and from 59.5 % to 116.5 % in culture medium. *R* was generally more variable in culture medium and lower than in Milli-Q for most amino acids, except for asparagine, aspartic acid, glutamine, histidine, glycine, arginine and methionine (Table S2). The precision of the method was determined by calculating the relative standard deviation (RSD %) for the replicates. Limits of detection (LOD) and quantification (LOQ) were determined from five replicates and defined as signal-to-noise ratios of ≥ 3 and ≥10, respectively. Replicates were measured in different runs in order to capture the variability observed between runs. LOD and LOQ could only be determined in Milli-Q due to the amino acid background of spent culture medium (Table S2). The high LOD and LOQ of alanine are likely due to variable levels of alanine contamination in Milli-Q and/or from the air. Lysine could not be reliably quantified in most runs.

**SI Results and Discussion**

**DFAA release by three *Nitrosopumilus* species under H_2_O_2_ stress**

Axenic cultures of *Nitrosopumilus adriaticus* NF5, *Nitrosopumilus piranensis* D3C and *Nitrosopumilus maritimus* SCM1 were grown in SCM medium without addition of purified catalase which resulted in growth retardation and inhibition due to accumulation of H_2_O_2_ (Bayer *et al.*, 2019). In cultures exposed to H_2_O_2_, the extracellular DFAA composition changed, resulting in an absolute increase in threonine and glutamine concentrations in the culture media of all three strains, as well as in a relative increase of histidine in *N. adriaticus* NF5 and *N. maritimus* SCM1 (Figure S2, Table S3). Exposure to H_2_O_2_ was accompanied by a stagnation in growth as previously reported (Bayer *et al.*, 2019). However, cell abundances did not decrease over the time course of the experiment, suggesting that increased release is not associated with cell death and biomass decay (Table S3). The increased release of DFAA could be due to membrane re-arrangement as a result of oxidative stress as described elsewhere (Bayer *et al.*, 2019), potentially leading to increased leakage of cytosolic metabolites. Alternatively, amino acids could be derived directly from the re-arranged membrane proteins since polar amino acids, generally found on the surface of a protein, were increasingly released under oxidative stress. However, the potentially non-efficient regulation of substrate uptake during stress conditions could also result in the secretion of non-metabolizable intermediates including amino acids.

**SI References**

Bayer, B., Pelikan, C., Bittner, M.J., Reinthaler, T., Könneke, M., Herndl, G.J., and Offre, P. (2019) Proteomic Response of Three Marine Ammonia-Oxidizing Archaea to Hydrogen Peroxide and Their Metabolic Interactions with a Heterotrophic Alphaproteobacterium. *mSystems* **4**: e00181-19.

Oksanen, J., Blanchet, F.G., Kindt, R., Legendre, P., Minchin, P.R., Mcglinn, D., et al. (2013) vegan: Community Ecology Package.

R Core Team (2013) R: A language and environment for statistical computing. R Foundation for Statistical Computing, Vienna, Austria. http://www.R-project.org.

SI Figures and Tables

**Figure S1** Principal coordinates analysis (PCoA, = Multidimensional scaling, MDS) of all resolved masses of compounds detected in *Nitrosopumilus* strains (*N. piranensis* D3C, *N. adriaticus* NF5 and *N. maritimus* SCM1), as well as in all medium blanks. Biological replicates are represented by different color shadings and technical replicates are represented by identical colors.

**Figure S2** Mean individual cell-specific DFAA production ratios of stressed vs non-stressed *Nitrosopumilus* strains (amol cell^-1^: amol cell^-1^) at different time points during early to late exponential growth phase (bars from left to right). Values above 1 indicate higher cell-specific production under stress conditions, whereas values below 1 indicate higher cell-specific DFAA production under optimal conditions.

**Table S1.** Elution gradient applied for the separation of dissolved free primary amino acids by HPLC. A: 89.8 % Methanol, 10 % Milli-Q water, 0.2 % Trifluoroacetic acid; B: Tetrahydrofuran; C: 40 mM NaH_2_PO_4_, pH 7.8

| **Elution time [min]** | **Eluent [%]** | | |
| --- | --- | --- | --- |
|  | **A** | **B** | **C** |
| 0 | 10 | 0 | 90 |
| 2 | 7.5 | 2.5 | 90 |
| 40 | 30 | 2 | 68 |
| 42 | 35 | 1 | 64 |
| 75 | 65 | 0 | 35 |
| 77 | 100 | 0 | 0 |
| 80 | 10 | 0 | 90 |

**Table S2**. Parameters for validation of the HPLC method used to measure dissolved free amino acid (DFAA) concentrations in the presence of high ammonium concentrations. ^‡^ The limits of detection (LOD) and quantification (LOQ) were determined in Milli-Q, due to presence of background concentrations of amino acids in the culture medium test samples (see SI Material and Methods).

| **Substance** | **Linearity** | **R^2^** | **LOD^‡^** | **LOQ^‡^** | **Recovery in MQ (n = 5; %)** | | | **Recovery in culture medium (n = 5; %)** | | |
| --- | --- | --- | --- | --- | --- | --- | --- | --- | --- | --- |
|  | **[nM]** |  | **[nM]** | **[nM]** | **[nM]** | | | **[nM]** | | |
|  |  |  |  |  | **5** | **50** | **500** | **5** | **50** | **500** |
| Aspartic acid | 1-1000 | 0.997-1.000 | ≤ 1 | < 5 | 92.1±8.0 | 97.4±3.6 | 99.0±4.3 | 93.4±2.8 | 83.9±5.2 | 86.2±5.5 |
| Glutamic acid | 1-1000 | 0.997-1.000 | < 1 | < 1 | 100.5±.3.7 | 94.9±2.6 | 94.3±2.3 | 86.4±2.2 | 72.9±3.7 | 73.3±4.3 |
| Asparagine | 1-100 | 0.996-1.000 | < 1 | < 1 | 96.1±.6.1 | 94.5±3.7 | 92.7±3.1 | 116.8±1.2 | 103.1±2.0 | 100.6±3.4 |
| Serine | 1-1000 | 0.998-1.000 | ≤ 1 | ≤ 5 | 107.5±3.5 | 108.3±3.4 | 106.3±2.0 | 66.7±2.3 | 72.1±3.8 | 78.5±2.0 |
| Glutamine | 1-1000 | 0.998-1.000 | < 1 | < 1 | 110.7±4.7 | 108.1±4.2 | 105.8±2.5 |  | 91.6±3.27 | 99.1±5.3 |
| Histidine | 1-1000 | 0.999 | < 1 | < 5 | 98.0±8.5 | 98.8±5.6 | 100.6±2.8 |  | 119.0±1.1 | 111.6±2.4 |
| Glycine | 1-1000 | 0.998-0.999 | ≤ 1 | < 5 | 103.9±1.4 | 107.0±1.0 | 107.2±3.2 |  | 103.2±0.8 | 95.9±0.1 |
| Threonine | 1-1000 | 0.999 | < 1 | ≤ 5 | 106.3±2.8 | 102.8±6.0 | 103.0±3.1 | 79.5±8.6 | 116.5±1.0 | 87.3±1.8 |
| Arginine | 1-1000 | 0.997-0.999 | < 1 | 5 | 93.5±8.7 | 106.9±2.3 | 105.7±4.8 |  | 112.4±1.5 | 94.6±2.6 |
| Alanine | 1-1000 | 0.997-0.999 | ≤ 5 | 10 | 109.0±1.3 | 107.3±2.9 | 106.3±2.0 |  | 84.7±2.9 | 77.7±4.2 |
| Tyrosine | 1-1000 | 0.996-1.000 | < 1 | < 5 | 111.3±4.3 | 107.3±3.6 | 106.9±2.3 | 84.4±3.5 | 76.8±2.9 | 82.9±4.2 |
| Valine | 1-1000 | 0.993-1.000 | 1 | < 5 | 110.2±2.1 | 106.9±3.2 | 104.7±2.8 |  | 69.0±4.3 | 68.8±5.3 |
| Methionine | 5-1000 | 0.989-1.000 | ≤ 1 | ≤ 5 | 115.4±5.2 | 115.1±3.0 | 111.5±3.2 |  | 109.7±3.6 | 105.0±2.0 |
| Tryptophan | 1-500 | 0.993-1.000 | ≤ 1 | ≤ 5 | 108.5±4.0 | 107.6±2.1 | 107.0±1.4 | 102.3±4.3 | 85.3±3.1 | 87.3±3.3 |
| Phenylalanine | 1-1000 | 0.991-1.000 | < 1 | < 1 | 110.7±2.6 | 108.0±3.3 | 106.6±2.0 |  | 82.4±0.4 | 80.6±4.2 |
| Isoleucine | 1-1000 | 0.995-1.000 | < 5 | < 5 | 109.3±3.3 | 106.3±3.0 | 105.9±2.3 |  | 59.5±5.3 | 69.6±6.3 |
| Leucine | 1-1000 | 0.995-1.000 | < 1 | < 5 | 111.3±1.4 | 111.0±2.8 | 107.9±1.3 | 76.8±4.5 | 67.3±3.1 | 73.4±4.3 |
| Lysine | 10-1000 | 0.988-0.999 | ≤ 1 | < 50 |  | 97.4±7.5 | 101.9±2.2 |  | 72.2±4.9 | 70.6±1.1 |

**Table S3**. Mean dissolved free amino acid (DFAA) concentrations (nM) produced by three *Nitrosopumilus* strains (*N. piranensis* D3C, *N. adriaticus* NF5 and *N. maritimus* SCM1). Standard deviations (≥1) of triplicate measurements are shown in parentheses. CAT, Catalase addition; bd, below detection; na, not available

| **Strain** | **CAT** | **Time (d)** | **Cells x10^6^**  **(ml^-1^)** | **Asp** | **Glu** | **Asn** | **Ser** | **Gln** | **His** | **Gly** | **Thr** | **Arg** | **Ala** | **Val^#^** | **Met^#^** | **Trp** | **Phe** | **Ile** | **Leu** | **Lys** |
| --- | --- | --- | --- | --- | --- | --- | --- | --- | --- | --- | --- | --- | --- | --- | --- | --- | --- | --- | --- | --- |
|  |  |  |  |  |  |  |  |  |  |  |  |  |  |  |  |  |  |  |  |  |
| NF5 | - | 0 | 3.1 |  |  |  |  |  |  |  |  |  |  |  |  |  |  |  |  |  |
| NF5 | - | 3 | 6.4 | 1 (1) | 4 (1) | 2 | 4 (1) | 4 | 12 (4) | 8 (4) | 23 (12) | bd | bd | na | na | bd | bd | bd | bd | bd |
| NF5 | - | 5 | 6.8 | 2 | 5 (1) | 3 | 11 (6) | 9 (2) | 17 (1) | 35 (2) | 37 (10) | bd | 5 (3) | na | na | bd | bd | 6 (9) | bd | bd |
| NF5 | - | 7 | 6.4 | 2 | 7 (1) | 2 | 17 (7) | 18 (9) | 18 (3) | 64 (6) | 36 (3) | bd | 9 (5) | na | na | bd | 6 (1) | 7 (3) | 3 | 2 (2) |
| NF5 | - | 9 | 6.8 | 3 (1) | 12 (1) | bd | 18 (9) | 16 (2) | 20 (2) | 86 (10) | 37 (2) | bd | 11 (7) | na | na | bd | 9 (2) | 10 (1) | 6 (1) | 8 (1) |
| NF5 | + | 0 | 3.1 |  |  |  |  |  |  |  |  |  |  |  |  |  |  |  |  |  |
| NF5 | + | 3 | 10.7 | bd | 2 | bd | 2 (1) | 2 | 5 (2) | 2 (1) | 8 (7) | bd | bd | na | na | bd | bd | 5 (5) | 2 (2) | bd |
| NF5 | + | 5 | 23.5 | 1 (1) | 4 | 2 | 9 (2) | 4 | 4 | 25 (2) | 11 (1) | bd | 34 (9) | na | na | bd | 13 (4) | 10 (3) | 15 (2) | 4 (1) |
| NF5 | + | 7 | 31.1 | 1 (1) | 5 | 3 | 17 (6) | 5 | 4 (2) | 49 (5) | 2 (2) | 3 (3) | 34 (4) | 45 (3) | bd | bd | 15 (3) | 4 (1) | 13 (2) | 10 (5) |
| NF5 | + | 9 | 34.6 | 2 (1) | 6 (1) | 2 | 20 (7) | 5 (1) | 6 (3) | 74 (6) | 4 (3) | 4 (3) | 33 (4) | 44 (2) | 2 | 3 (1) | 15 (1) | 4 (1) | 12 (1) | 24 (5) |
| D3C | - | 0 | 2.1 |  |  |  |  |  |  |  |  |  |  |  |  |  |  |  |  |  |
| D3C | - | 3 | 3.5 | 1 | 3 (1) | 5 (3) | 1 (1) | 4 (1) | bd | 2 (1) | 5 (2) | bd | 4 (3) | na | na | bd | 9 (4) | 3 (2) | bd | na |
| D3C | - | 6 | 4.4 | 2 | 7 | 20 (3) | 2 (1) | 22 (5) | bd | 16 | 20 (2) | bd | 5 (2) | na | na | bd | 17 (3) | 2 (2) | bd | na |
| D3C | - | 9 | 4.3 | 4 (1) | 9 (2) | 29 (6) | 5 (1) | 38 (7) | bd | 35 (3) | 23 (5) | bd | 8 (1) | na | na | 4 (2) | 27 (6) | 15 (6) | bd | na |
| D3C | + | 0 | 2.1 |  |  |  |  |  |  |  |  |  |  |  |  |  |  |  |  |  |
| D3C | + | 3 | 5 | 2 (2) | 5 (6) | 3 (1) | 2 (3) | 2 (2) | bd | 5 (6) | 2 (3) | bd | 3 (3) | na | na | bd | 16 (11) | 2 (3) | 2 (2) | na |
| D3C | + | 6 | 11.7 | 2 | 7 (1) | 10 (2) | 2 | 7 (2) | bd | 21 (1) | 9 | bd | 48 (4) | na | na | 7 (5) | 16 (9) | 9 (2) | 16 (1) | na |
| D3C | + | 9 | 29.4 | 5 (1) | 17 (3) | 21 (1) | 6 (2) | 9 (2) | bd | 121 (5) | 5 (4) | bd | 82 (12) | 66 (12) | 42 (21) | 10 (9) | 25 (2) | 17 (5) | 28 (5) | na |
| SCM1 | - | 0 | 1.8 |  |  |  |  |  |  |  |  |  |  |  |  |  |  |  |  |  |
| SCM1 | - | 3 | 4.1 | bd | 2 | 5 (1) | bd | 6 (3) | 2 (2) | 3 (4) | 12 (3) | bd | bd | na | na | bd | bd | bd | bd | na |
| SCM1 | - | 6 | 10.5 | 1 (1) | 7 (1) | 15 (2) | 3 (1) | 24 (6) | 3 (3) | 22 (2) | 23 (3) | bd | 2 | na | na | bd | bd | 3 (3) | 3 (3) | na |
| SCM1 | - | 8 | 10.4 | 2 | 9 (2) | 24 (1) | 6 (2) | 28 (6) | 3 (3) | 46 (4) | 29 (2) | bd | 9 (5) | na | na | bd | 3 (1) | 5 (4) | 6 (1) | na |
| SCM1 | + | 0 | 1.8 |  |  |  |  |  |  |  |  |  |  |  |  |  |  |  |  |  |
| SCM1 | + | 3 | 5.2 | 1 | 1 | 4 (1) | 1 (1) | bd | 2 (2) | 1 (2) | 3 (3) | bd | 5 (8) | na | na | bd | bd | bd | bd | na |
| SCM1 | + | 6 | 29.4 | 2 (1) | 9 (2) | 12 (2) | 4 (2) | 3 (2) | 3 (3) | 24 (4) | 11 | 2 (2) | 55 (27) | 50 (2) | 25 (8) | 2 | 17 (9) | 3 (2) | 21 (9) | na |
| SCM1 | + | 8 | 48.2 | 3 | 12 | 16 (1) | 5 | 3 (1) | 5 (1) | 88 (2) | na | 7 (2) | 46 (8) | 56 (3) | 29 (9) | 2 (1) | 22 (3) | 5 (2) | 21 (1) | na |

**^#^** Val and Met could only be quantified in the absence of NH_4_^+^/NH_3_, corresponding to the end of exponential growth under optimal conditions. Thus, T0 measurements could not be subtracted.

**Table S4.** Comparison of intracellular DFAA of three *Nitrosopumilus* strains (*N. piranensis* D3C, *N. adriaticus* NF5 and *N. maritimus* SCM1) and the average amino acid composition encoded by the genomes. Values are given in mol%.

| **Amino acid** | **Genome average** | **D3C** | **NF5** | **SCM1** |
| --- | --- | --- | --- | --- |
| Ala | 5.76 | 5.18 | 6.98 | 6.19 |
| Arg | 3.37 | 2.24 | 5.35 | 3.72 |
| Asn | 5.09 | 2.62 | 2.24 | 2.69 |
| Asp | 6.04 | 6.04 | 2.86 | 5.19 |
| Gln | 3.15 | 9.53 | 3.12 | 7.10 |
| Glu | 7.07 | 40.09 | 39.18 | 36.28 |
| Gly | 6.38 | 1.84 | 1.45 | 4.23 |
| His | 1.78 | 1.14 | 2.29 | 1.12 |
| Ile | 9.10 | 2.97 | 3.87 | 3.40 |
| Leu | 8.60 | 2.61 | 1.74 | 2.76 |
| Lys | 8.64 | 3.56 | 7.32 | 5.82 |
| Met | 2.50 | 0.97 | 0.51 | 1.23 |
| Phe | 4.46 | 1.32 | 1.14 | 1.78 |
| Ser | 7.42 | 4.76 | 5.64 | 6.61 |
| Thr | 5.54 | 6.12 | 4.41 | 4.88 |
| Trp | 0.89 | 0.62 | 0.81 | 0.64 |
| Tyr | 3.06 | 3.42 | 5.99 | 3.02 |
| Val | 6.40 | 4.95 | 5.11 | 3.34 |
| Pro | 3.74 | na | na | na |
| Cys | 1.01 | na | na | na |
